# Genome sequencing and analysis of *Ralstonia solanacearum* phylotype I strains FJAT-91, FJAT-452 and FJAT-462 isolated from tomato, eggplant, and chili pepper in China

**DOI:** 10.1186/s40793-017-0241-7

**Published:** 2017-04-17

**Authors:** Yidan Sun, Keke Wang, Carlos Caceres-Moreno, Wei Jia, Aojun Chen, Heng Zhang, Renyi Liu, Alberto P. Macho

**Affiliations:** 1 Shanghai Center for Plant Stress Biology, CAS Center for Excellence in Molecular Plant Sciences; Shanghai Institutes of Biological Sciences, Chinese Academy of Sciences, Shanghai, 201602 China; 2 University of Chinese Academy of Sciences, Beijing, 100039 China

**Keywords:** *Ralstonia*, Virulence, Effector, Tomato, Eggplant, Chili pepper, China

## Abstract

*Ralstonia solanacearum* is an extremely destructive pathogen able to cause disease in a wide range of host plants. Here we report the draft genome sequences of the strains FJAT-91, FJAT-452 and FJAT-462, isolated from tomato, eggplant, and chili pepper, respectively, in China. In addition to the genome annotation, we performed a search for type-III secreted effectors in these strains, providing a detailed annotation of their presence and distinctive features compared to the effector repertoire of the reference phylotype I strain (GMI1000). In this analysis, we found that each strain has a unique effector repertoire, encoding both strain-specific effector variants and variations shared among all three strains. Our study, based on strains isolated from different hosts within the same geographical location, provides insight into effector repertoires sufficient to cause disease in different hosts, and may contribute to the identification of host specificity determinants for *R. solanacearum*.

## Introduction

*Ralstonia solanacearum* is often considered one of the most destructive bacterial pathogens, causing bacterial wilt disease in more than 250 plant species worldwide [1]. The pathogenicity of *R. solanacearum* heavily relies on the injection of proteins inside plant cells through a type-III secretion system (T3SS). The versatility of *R. solanacearum* strains correlates with the presence of a larger number of T3SS substrates, called type-III effectors (T3Es), encoded in their genomes, in comparison to other bacterial pathogens [2]. T3Es are important virulence factors required by most gram-negative pathogens to manipulate plant cells and cause disease [3, 4]. Bacteria from a single *R. solanacearum* strain can inject more than 70 T3Es (termed Rips for *Ralstonia* injected proteins) inside plant cells [2, 5]. Studies conducted in *Pseudomonas syringae* and *Xanthomonas axonopodis* strains indicate that T3E repertoires are highly variable among strains of these species, and led to the hypothesis that T3E composition may shape the host range of bacterial pathogens [6, 7]. Although the genome sequences and T3E repertoires have been defined for several *R. solanacearum* strains, repertoire comparisons have failed in identifying host specificity determinants so far [2], which may suggest that genome sequences from additional strains infecting different hosts are required for this analysis. Additionally, the diversity in the geographical origins of sequenced strains hinders this comparative analysis, since additional environmental factors, such as temperature, light, and humidity may have a significant impact on the requirement of effectors for a successful infection. In this project, we sequenced and annotated the genomes of the *R. solanacearum* strains FJAT-91, FJAT-452 and FJAT-462, isolated from tomato (*Solanum lycopersicum*), eggplant (*Solanum melongena*), and chili pepper (*Capsicum* *annum*), respectively, in the Fujian province (China) [8]. In addition, we performed a search for T3Es in these strains, providing a detailed annotation of their presence and distinctive features compared to the effector repertoire of the reference phylotype I strain (GMI1000). To our knowledge, this is the first report of genome sequences combined with T3E repertoire analysis performed in strains isolated from different hosts with the same geographical origin.

## Organism information

### Classification and features

**Fig. 1:**
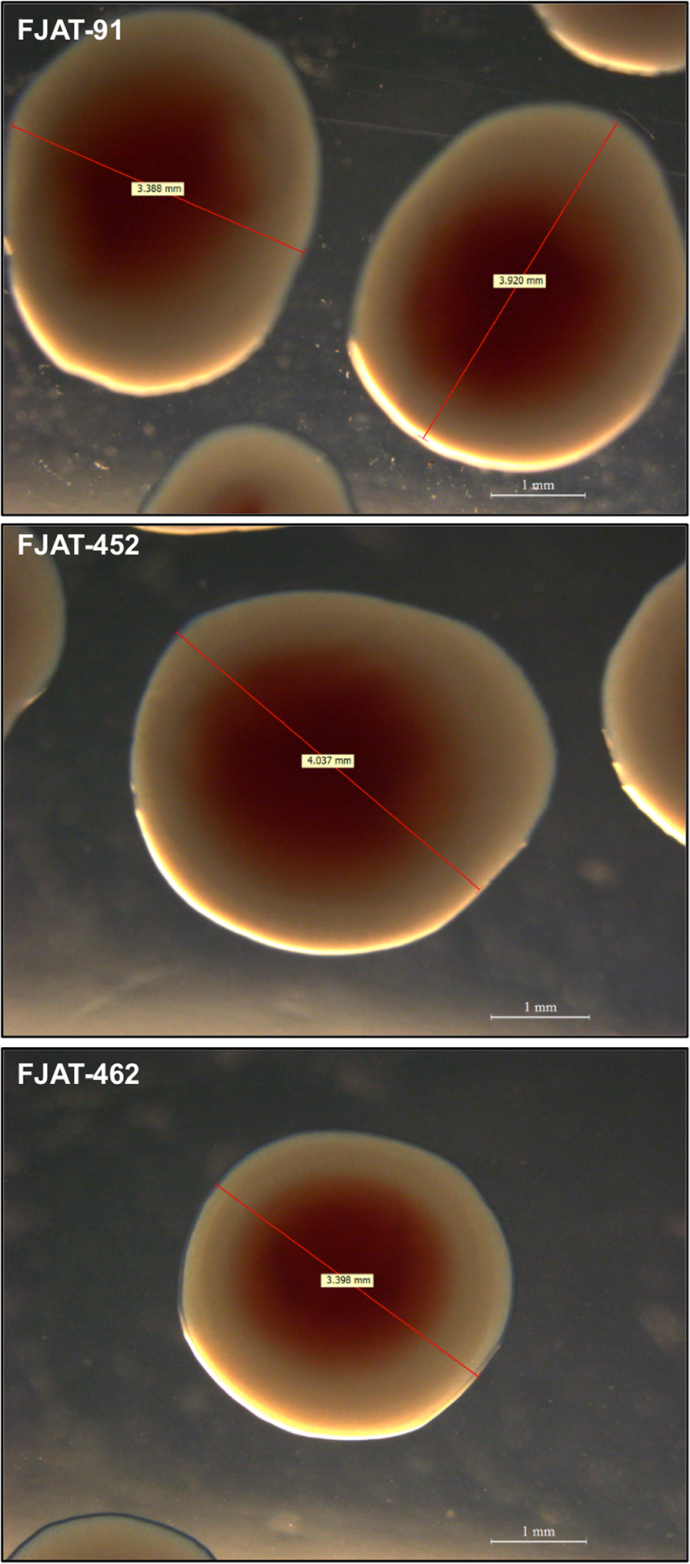
Images of the *Ralstonia solanacearum* strains using a stereo microscope to visualize colony morphology on solid medium. The strains were grown on rich medium at 28 °C for 2 days. Scale bars (1 mm) and the size of representative colonies are provided for reference

**Fig. 2:**
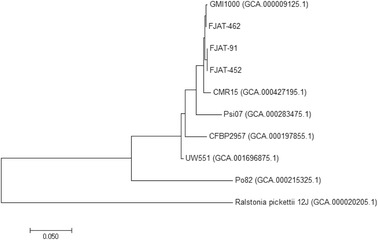
Phylogenetic tree showing the position of the *Ralstonia solanacearum* sequenced in this study, relative to other sequenced strains from the same species. The phylogenetic tree was constructed using concatenated alignments of the marker genes *fliC*, *hrpB* and *pehA*. The evolutionary history was inferred by using the Maximum Likelihood method based on the Tamura-Nei model [27]. Evolutionary analyses were conducted in MEGA7 [28]. GenBank accession numbers are displayed within brackets. *Ralstonia pickettii* 12 J was used as an outgroup

*Ralstonia solanacearum* belongs to the order *Burkholderiales* of the class *Betaproteobacteria*. It is an aerobic, Gram-negative bacterium, naturally present in soil, water, infected plants or plant debris. It has a worldwide distribution, with higher incidence in tropical and subtropical regions, but also present in other temperate areas [9]. *R. solanacearum* is the agent causing bacterial wilt disease in multiple host plants, characterized by a sudden wilt of the whole plant. The strains sequenced in this study, FJAT-91, FJAT-452 and FJAT-462, were isolated from naturally infected tomato (*Solanum lycopersicum*), eggplant (*Solanum melongena*), and chili pepper (*Capsicum* *annum*) plants, respectively, in the Fujian province (China). Plants showing typical wilting symptoms were collected, surface-sterilized, and the tissue was homogenized with sterile water before plating serial dilutions to determine the causal agent [8, 9]. Sequence analysis determined that they belong to the *R. solanacearum* species complex [8]. The pathogenicity of FJAT-91 has been confirmed and used as positive control for pathogenicity assays in tomato plants in previous studies [10]. All three isolated strains displayed the typical physiological features of strains from the *R. solanacearum* species complex, showing aerobic growth in laboratory conditions, and were able to form 3–4 mm colonies within 2 days at 28 °C when grown on a rich laboratory medium containing tetrazolium chloride and high glucose content. For all three strains, colony shape was irregular, mucooid, and displayed a pink area in the middle of the colony and a large white edge (Fig. 1). Gene sequence analysis of PCR-amplified *fliC*, *hrpB* and *pehA* genes indicated that these strains belong to the phylotype I (represented by the reference strain GMI1000; Fig. 2), mostly formed by Asian strains [2]. The classification and general features of the three strains are summarized in the Tables 1, 2 and 3, and a phylogenetic tree is shown in the Fig. 2.

**Table 1:** Classification and general features of *Ralstonia solanacearum* FJAT-91 strain according to the MIGS recommendations [29]

MIGS ID	Property	Term	Evidence code^a^
	Classification	Domain *Bacteria*	TAS [30]
		Phylum *Proteobacteria*	TAS [31]
		Class *Betaproteobacteria*	TAS [32, 33]
		Order *Burkholderiales*	TAS [32, 33]
		Family *Burkholderiaceae*	TAS [32, 33]
		Genus *Ralstonia*	TAS [34, 35]
		Species *Ralstonia solanacearum*	TAS [34, 35]
		Strain: *FJAT-91*	
	Gram stain	Negative	IDA
	Cell shape	Rod	IDA
	Motility	Motile	IDA
	Sporulation	Non sporulating	NAS
	Temperature range	Mesophile	IDA
	Optimum temperature	27 °C	IDA
	pH range; Optimum	5.5–8.0; 6.5	NAS
	Carbon source	Dextrose, lactose, maltose, cellobiose	IDA
MIGS-6	Habitat	Tomato plants. Soil.	TAS [8, 10]
MIGS-6.3	Salinity	<2.0%	TAS [36]
MIGS-22	Oxygen requirement	Aerobic	IDA
MIGS-15	Biotic relationship	free-living	IDA
MIGS-14	Pathogenicity	Pathogenic	TAS [8, 10]
MIGS-4	Geographic location	Fuzhou, Fujian, China	TAS [8]
MIGS-5	Sample collection	2011	TAS [8]
MIGS-4.1	Latitude	26°4'28.23"N,	NAS
MIGS-4.2	Longitude	119°17'47.38"E	NAS
MIGS-4.4	Altitude	9.74 m	NAS

^a^Evidence codes - IDA: Inferred from Direct Assay; TAS: Traceable Author Statement (i.e., a direct report exists in the literature); NAS: Non-traceable Author Statement (i.e., not directly observed for the living, isolated sample, but based on a generally accepted property for the species, or anecdotal evidence). These evidence codes are from the Gene Ontology project [37]

**Table 2:** Classification and general features of *Ralstonia solanacearum* FJAT-452 strain according to the MIGS recommendations [29]

MIGS ID	Property	Term	Evidence code^a^
	Classification	Domain *Bacteria*	TAS [30]
		Phylum *Proteobacteria*	TAS [31]
		Class *Betaproteobacteria*	TAS [32, 33]
		Order *Burkholderiales*	TAS [32, 33]
		Family *Burkholderiaceae*	TAS [32, 33]
		Genus *Ralstonia*	TAS [34, 35]
		Species *Ralstonia solanacearum*	TAS [34, 35]
		Strain: *FJAT-452*	
	Gram stain	Negative	IDA
	Cell shape	Rod	IDA
	Motility	Motile	IDA
	Sporulation	Non sporulating	NAS
	Temperature range	Mesophile	IDA
	Optimum temperature	27 °C	IDA
	pH range; Optimum	5.5–8.0; 6.5	NAS
	Carbon source	Dextrose, lactose, maltose, cellobiose	IDA
MIGS-6	Habitat	Eggplants. Soil.	TAS [8, 10]
MIGS-6.3	Salinity	<2.0%	TAS [36]
MIGS-22	Oxygen requirement	Aerobic	IDA
MIGS-15	Biotic relationship	free-living	IDA
MIGS-14	Pathogenicity	Pathogenic	TAS [8, 10]
MIGS-4	Geographic location	Ningde, Fujian, China.	TAS [8]
MIGS-5	Sample collection	2011	TAS [8]
MIGS-4.1	Latitude	26°39'56.22"N	NAS
MIGS-4.2	Longitude	119°32'52.56"E	NAS
MIGS-4.4	Altitude	2.89 m	NAS

^a^Evidence codes - IDA: Inferred from Direct Assay; TAS: Traceable Author Statement (i.e., a direct report exists in the literature); NAS: Non-traceable Author Statement (i.e., not directly observed for the living, isolated sample, but based on a generally accepted property for the species, or anecdotal evidence). These evidence codes are from the Gene Ontology project [37]

**Table 3:** Classification and general features of *Ralstonia solanacearum* FJAT-462 strain according to the MIGS recommendations [29]

MIGS ID	Property	Term	Evidence code^a^
	Classification	Domain *Bacteria*	TAS [30]
		Phylum *Proteobacteria*	TAS [31]
		Class *Betaproteobacteria*	TAS [32, 33]
		Order *Burkholderiales*	TAS [32, 33]
		Family *Burkholderiaceae*	TAS [32, 33]
		Genus *Ralstonia*	TAS [34, 35]
		Species *Ralstonia solanacearum*	TAS [34, 35]
		Strain: *FJAT-462*	
	Gram stain	Negative	IDA
	Cell shape	Rod	IDA
	Motility	Motile	IDA
	Sporulation	Non sporulating	NAS
	Temperature range	Mesophile	IDA
	Optimum temperature	27 °C	IDA
	pH range; Optimum	5.5–8.0; 6.5	NAS
	Carbon source	Dextrose, lactose, maltose, cellobiose	IDA
MIGS-6	Habitat	Chili pepper plants. Soil.	TAS [8, 10]
MIGS-6.3	Salinity	<2.0%	TAS [36]
MIGS-22	Oxygen requirement	Aerobic	IDA
MIGS-15	Biotic relationship	free-living	IDA
MIGS-14	Pathogenicity	Pathogenic	TAS [8, 10]
MIGS-4	Geographic location	Ningde, Fujian, China	TAS [8]
MIGS-5	Sample collection	2011	TAS [8]
MIGS-4.1	Latitude	26°39'56.22"N	NAS
MIGS-4.2	Longitude	119°32'52.56"E	NAS
MIGS-4.4	Altitude	2.89 m	NAS

^a^Evidence codes - IDA: Inferred from Direct Assay; TAS: Traceable Author Statement (i.e., a direct report exists in the literature); NAS: Non-traceable Author Statement (i.e., not directly observed for the living, isolated sample, but based on a generally accepted property for the species, or anecdotal evidence). These evidence codes are from the Gene Ontology project [37]

## Genome sequencing information

### Genome project history

This sequencing project was started in 2015, assembly and annotation was performed in 2016. Assembled draft genome sequences for the strains FJAT-91, FJAT-452 and FJAT-462 have been deposited to GenBank (Table 4). Raw genomic reads have been deposited to the Sequence Read Archive with accession numbers SRP091690, SRR4431158, SRR4431159, SRR4428740.

**Table 4:** Project information

MIGS ID	Property	FJAT-91	FJAT-452	FJAT-462
MIGS 31	Finishing quality	Draft	Draft	Draft
MIGS 28	Libraries used	Vazyme TruePrep PE250	Vazyme TruePrep PE250	Vazyme TruePrep PE250
MIGS 29	Sequencing platforms	Illumina HiSeq 2500	Illumina HiSeq 2500	Illumina HiSeq 2500
MIGS 31.2	Fold coverage	>50X	>50X	>50X
MIGS 30	Assemblers	SOAPdenovo2; GapCloser v1.12; CONTIGuator	SOAPdenovo2; GapCloser v1.12; CONTIGuator	SOAPdenovo2; GapCloser v1.12; CONTIGuator
MIGS 32	Gene calling method	Prokka v1.11 (ncRNAs search enabled)	Prokka v1.11 (ncRNAs search enabled)	Prokka v1.11 (ncRNAs search enabled)
	Locus Tag	NA	NA	NA
	Genbank ID	MLYU00000000	MLYS00000000	MLYT00000000
	GenBank Date of Release	December 25, 2016	December 25, 2016	December 25, 2016
	GOLD ID	NA	NA	NA
	BIOPROJECT	PRJNA347535	PRJNA347535	PRJNA347535
MIGS 13	Source Material Identifier	SAMN05892025	SAMN05892026	SAMN05892027
	Project relevance	Plant pathogen	Plant pathogen	Plant pathogen

### Growth conditions and genomic DNA preparation

*R. solanacearum* strains were grown in rich medium (10 g/l bactopeptone, 1 g/l yeast extract and 1 g/l casamino acids). Genomic DNA was extracted from bacterial cultures grown to stationary phase for 18 h at 28 °C and shaking at 220 rpm (OD_600_ = 1) using the Blood & Cell Culture DNA Mini kit (Qiagen), following manufacturer’s instructions for gram-negative bacteria. DNA concentration and quality were measured using a Qubit 2.0 Fluorometer (Invitrogen).

### Genome sequencing and assembly

For each genome, we prepared a paired-end library with an average insert size of 470 bp and sequenced the library for 250 bp from both ends using Illumina HiSeq 2500. The number of raw read bases was greater than 300 million (>50x genome coverage) for each sequenced strain. The raw sequencing data were first preprocessed to remove adapter sequences, low-quality regions, and short sequences (less than 20 nucleotides) with Cutadapt [11] and SolexaQA [12]. The remaining clean reads were *de novo* assembled into contigs and scaffolds by using SOAPdenovo2 and GapCloser v1.12 [13]. Contigs and scaffolds were further assembled into chromosome, plasmid and scaffolds with CONTIGuator, using the GMI1000 genome as the reference. The resulting FJAT-91, FJAT-452 and FJAT-462 genomes are 4,620,128 bp, 5,334,434 bp and 5,083,617 bp, respectively (Table 5), close to the genome length of the *R. solanacearum* reference strain GMI1000 (5,810,922 bp) [14].

**Table 5:** Genome and annotation statistics of the three newly sequenced *Ralstonia solanacearum* strains

Attribute	FJAT-91		FJAT-452		FJAT-462	
	Value	% of total	Value	% of total	Value	% of total
Genome size (bp)	4,620,128	100.00	5,334,434	100.00	5,083,617	100.00
DNA coding (bp)	3,003,037	65.00	3,696,229	69.29	3,397,556	66.83
DNA G + C (bp)	2,799,660	60.60	3,324,908	62.33	3,123,835	61.45
DNA scaffolds	329	100.00	309	100.00	358	100.00
Total genes	6522	100.00	6729	100.00	6758	100.00
Protein coding genes	6457	99.00	6658	98.94	6696	99.08
RNA genes	65	1.00	71	1.06	62	0.92
Pseudo genes	NA	NA	NA	NA	NA	NA
Genes in internal clusters	NA	NA	NA	NA	NA	NA
Genes with function prediction	2544	39.40	3075	46.19	2855	42.64
Genes assigned to COGs	2714	42.03	3263	49.01	3046	45.49
Genes with Pfam domains	2361	36.56	2948	44.28	2,674	39.93
Genes with signal peptides	270	4.18	334	5.02	303	4.53
Genes with transmembrane helices	291	4.51	349	5.24	311	4.64
CRISPR repeats	0	-	0	-	0	-

### Genome annotation

Genome annotation was performed using Prokka (v1.11) [15] with the option for non-coding RNA (ncRNA) search. The COG database [16] and Pfam v30.0 [17] were used for functional annotation of genes. T3Es in the three newly sequenced strains were identified and annotated in two steps: first, 52, 62 and 60 of the T3Es from the *R. solanacearum* species complex [2] were identified in FJAT-91, FJAT-452 and FJAT-462, respectively, based on Prokka annotations; second, known T3Es protein sequences [2] were used as query to search the assembled genome sequences of three strains using BLAST [18] with a stringent significance cutoff of e-value < 1e-30, identity > 60, and coverage on the query T3E protein sequence being over 50% or at least 100 aa in length. As a result, 72, 78 and 75 T3Es were identified in FJAT-91, FJAT-452 and FJAT-462, respectively. These two sets of T3E genes were merged together to generate the final lists of T3E genes in the three genomes. To identify the sequence variations within T3E genes between three strains and the reference strain, the clean reads from the three newly sequenced strains were mapped to the reference genome GMI1000 using BWA (v0.7.12) [19]. SNPs and INDELs were identified using Samtools (v0.1.19) [20] and vcftools (v0.1.12) [21] and were further annotated using SnpEff (v4.0) [22].

## Genome properties

The genome of *R. solanacearum* strain FJAT-91 has 329 scaffolds and the average GC content of the genome is 60.6% (Table 5). A total of 6,522 genes (6457 CDSs and 65 ncRNAs) were predicted. Of the protein-coding genes, 2544 (39.4%) had functions assigned while 3913 were considered hypothetical (Table 5). 42.03% of the CDSs could be assigned to one COG functional category and 36.56% contained one or more conserved PFAM-A domains (Table 6). The genome of *R. solanacearum* strain FJAT-452 has 309 scaffolds and the average GC content of the genome is 62.33% (Table 5). A total of 6729 genes (6658 CDSs and 71 ncRNAs) were predicted. Of the protein-coding genes, 3075 (46.19%) had functions assigned while 3583 were considered hypothetical (Table 5). 49.01% of the CDSs could be assigned to one COG functional category and 44.28% contained one or more conserved PFAM-A domains (Table 6). The genome of *R. solanacearum* strain FJAT-462 has 358 scaffolds and the average GC content of the genome is 61.45% (Table 5). A total of 6758 genes (6696 CDSs and 62 ncRNAs) were predicted. Of the protein-coding genes, 2855 (42.64%) had functions assigned while 3,841 were considered hypothetical (Table 5). 45.49% of the CDSs could be assigned to one COG functional category and 39.93% contained one or more conserved PFAM-A domains (Table 6).

**Table 6:** Number of genes that are associated with different COG functional categories

Code	FJAT-91		FJAT-452		FJAT-462		Description
	Value	% of total	Value	% of total	Value	% of total	
J	173	2.68%	189	2.84%	185	2.76%	Translation, ribosomal structure and biogenesis
A	2	0.03%	2	0.03%	2	0.03%	RNA processing and modification
K	221	3.42%	273	4.10%	264	3.94%	Transcription
L	104	1.61%	119	1.79%	115	1.72%	Replication, recombination and repair
B	2	0.03%	2	0.03%	3	0.04%	Chromatin structure and dynamics
D	47	0.73%	51	0.77%	53	0.79%	Cell cycle control, cell division, chromosome partitioning
V	52	0.81%	60	0.90%	61	0.91%	Defense mechanisms
T	167	2.59%	191	2.87%	180	2.69%	Signal transduction mechanisms
M	172	2.66%	217	3.26%	197	2.94%	Cell wall/membrane/envelope biogenesis
N	105	1.63%	116	1.74%	109	1.63%	Cell motility
Z	1	0.02%	1	0.02%	1	0.01%	Cytoskeleton
W	3	0.05%	3	0.05%	3	0.04%	Extracellular structures
U	107	1.66%	109	1.64%	119	1.78%	Intracellular trafficking, secretion, and vesicular transport
O	131	2.03%	144	2.16%	150	2.24%	Posttranslational modification, protein turnover, chaperones
X	65	1.01%	120	1.80%	71	1.06%	Mobilome: prophages, transposons
C	191	2.96%	231	3.47%	213	3.18%	Energy production and conversion
G	151	2.34%	185	2.78%	166	2.48%	Carbohydrate transport and metabolism
E	232	3.59%	288	4.33%	263	3.93%	Amino acid transport and metabolism
F	62	0.96%	77	1.16%	71	1.06%	Nucleotide transport and metabolism
H	107	1.66%	140	2.10%	127	1.90%	Coenzyme transport and metabolism
I	153	2.37%	193	2.90%	180	2.69%	Lipid transport and metabolism
P	131	2.03%	147	2.21%	143	2.14%	Inorganic ion transport and metabolism
Q	58	0.90%	69	1.04%	60	0.90%	Secondary metabolites biosynthesis, transport and catabolism
R	175	2.71%	213	3.20%	200	2.99%	General function prediction only
S	182	2.82%	232	3.48%	211	3.15%	Function unknown
-	3743	57.97%	3395	50.99%	3650	54.51%	Not assigned to any COG categories

The total is based on the total number of protein coding genes in the genome

## Insights from the genome sequence

### Comparative analysis of virulence-related genes

T3E proteins are essential virulence factors in most gram-negative bacterial pathogens, such as *R. solanacearum* [2, 5], although they can also be perceived by resistant hosts as invasion signals, leading the development of plant defense responses [23]. The expression of genes encoding T3Es and structural components of the T3SS is activated after the perception of plant signals, and coordinated by a well-studied signaling pathway [24]. We analyzed the presence of genes involved in plant sensing and virulence regulation in the newly sequenced strains, and found that all the major regulators are present in the three strains (Table 7). These genes displayed a high percentage of similarity when compared to their homologs in the GMI1000 reference strain, ranging from 98.97 to 100% at the DNA level and from 99.19 to 100% at the amino acid level (Table 7).

**Table 7:** Sequence similarity of genes involved in *R. solanacearum* virulence between the three newly sequenced strains and the reference stain GMI1000

	FJAT-91		FJAT-452		FJAT-462	
	Gene	Protein	Gene	Protein	Gene	Protein
Plant sensing and virulence regulation						
hrpG	99.46	99.59	99.46	99.59	99.32	99.19
prhA	99.54	99.77	99.75	100.00	99.62	100.00
prhJ	99.23	100.00	99.04	99.43	98.97	99.38
prhR	100.00	100.00	99.61	100.00	99.19	98.78
Non-T3SS virulence factors						
egl	99.05	99.37	99.12	99.20	98.94	99.20
epsA	99.56	100.00	99.74	100.00	99.83	100.00
epsB	99.63	99.86	99.58	99.86	99.58	99.86
epsC	99.82	99.74	99.82	99.74	99.82	99.74
epsD	99.68	100.00	99.68	100.00	99.68	100.00
epsE	99.40	100.00	99.54	99.77	99.62	99.77
epsF	99.75	99.75	99.68	100.00	98.35	98.26
epsP	99.77	100.00	99.77	100.00	99.77	100.00
epsR	99.86	100.00	99.86	100.00	99.86	100.00
pehB	99.05	99.43	99.68	99.04	99.15	99.15
phcA	99.62	99.71	99.62	99.71	99.71	99.71
phcB	86.41	85.84	86.41	85.84	99.93	100.00
vsrA	98.34	97.84	99.65	100.00	99.65	99.79
vsrB	99.89	100.00	99.57	100.00	99.40	99.67
vsrC	99.70	100.00	99.70	100.00	99.70	100.00
vsrD	100.00	100.00	100.00	100.00	100.00	100.00
xpsR	99.78	99.67	99.78	100.00	99.67	99.67

**Fig. 3:**
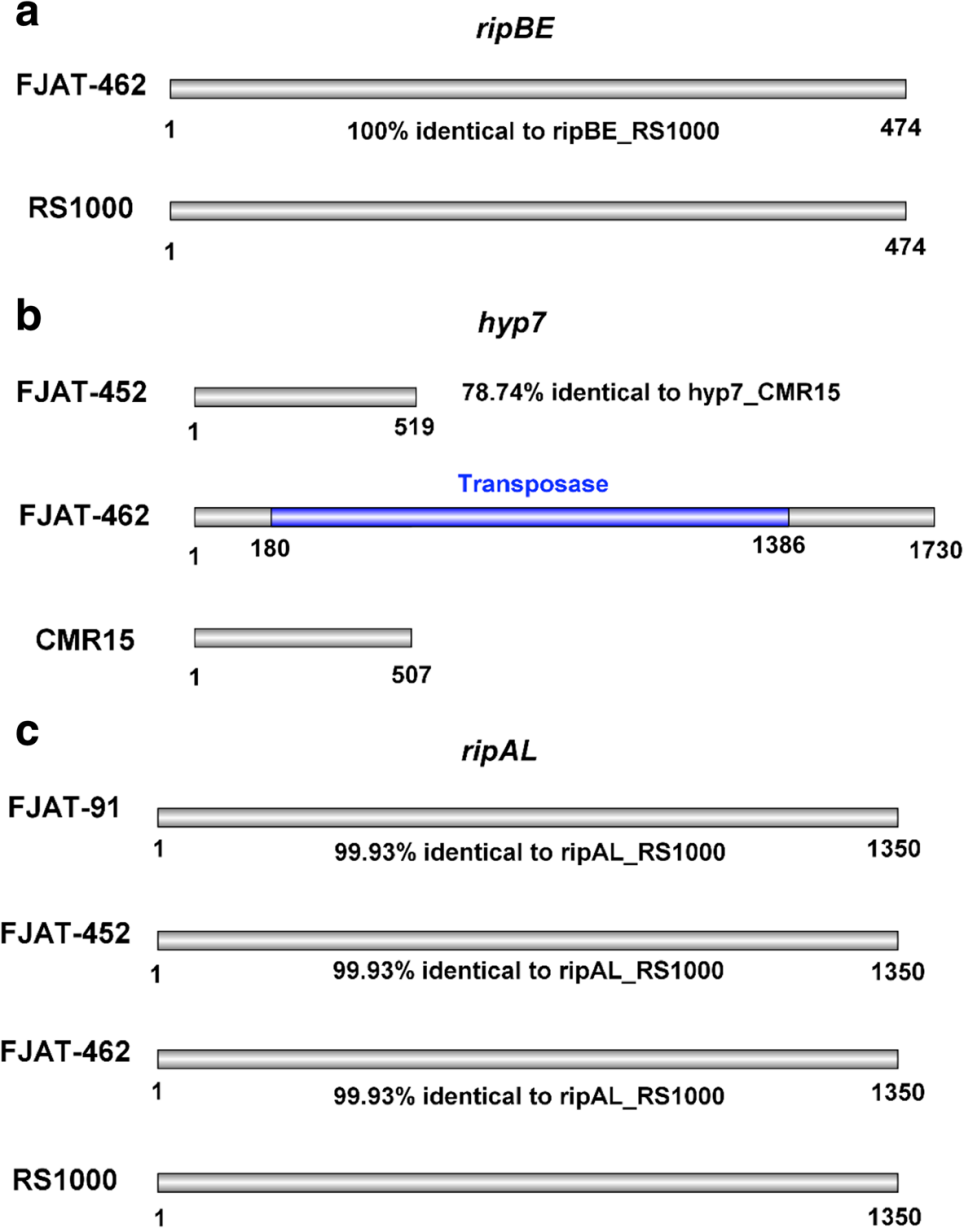
Schematic diagram of *ripBE* (**a**), *hyp7* (**b**) and *ripAL* (**c**) sequence alignment. The nucleotide sequence of these genes in the strains sequenced in this study is 100% identical to each other, except for *hyp7*, which has an insertion annotated as a transposase in FJAT-462 (numbers indicate the insertion site). The percentage of identity compared to the orthologs in other sequenced strains is indicated in the figure

**Fig. 4:**
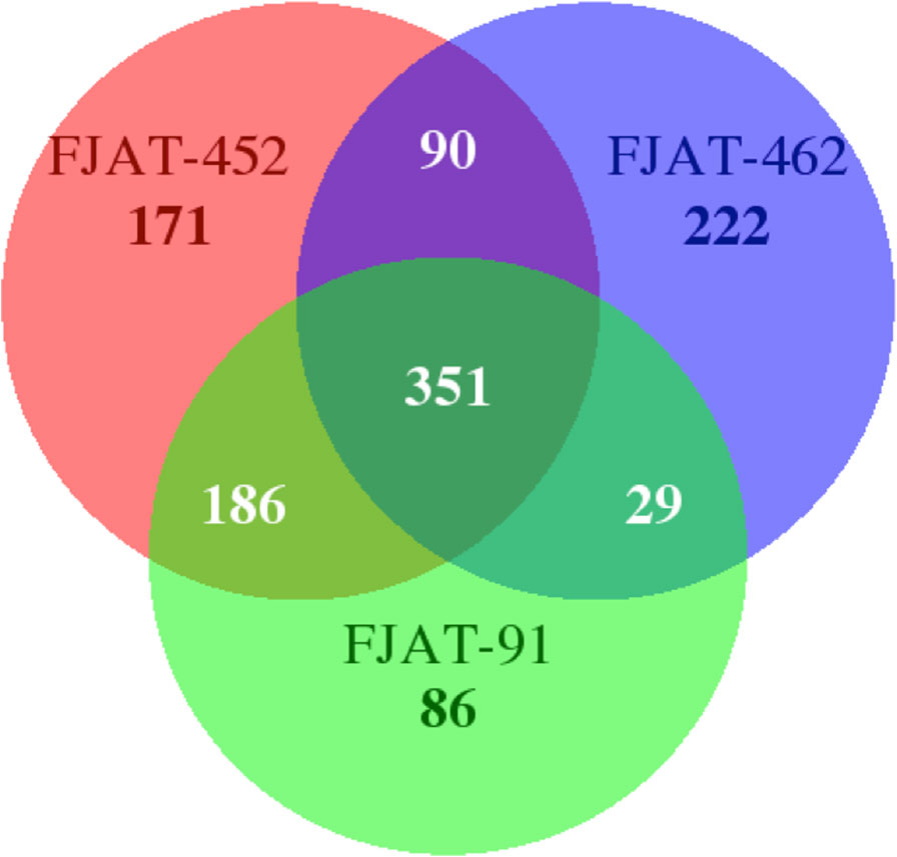
Venn diagram of T3E gene variants identified in FJAT-91, FJAT-452 and FJAT-462 when compared to the reference stain GMI1000

**Fig. 5:**
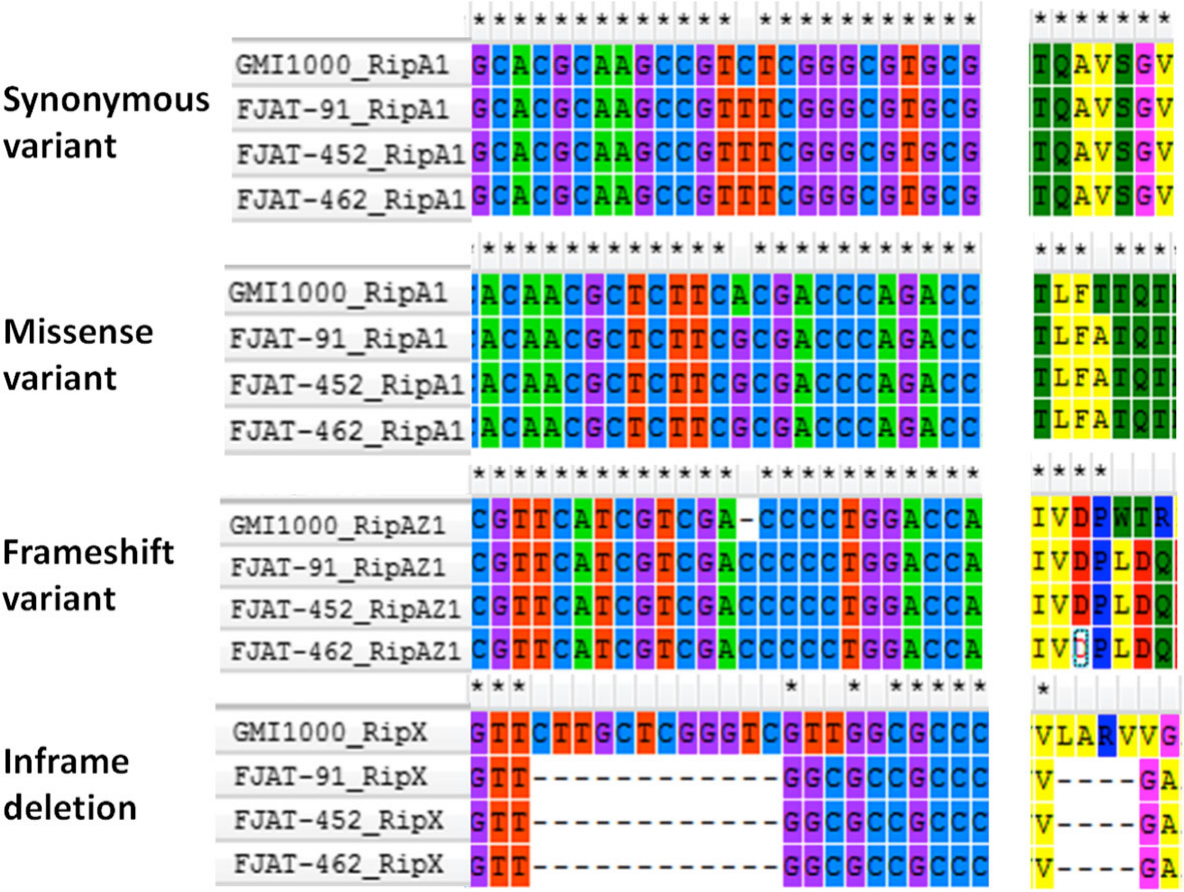
Examples of shared sequence variations in *ripA1*, *ripAZ1* and *ripX* genes among three newly sequenced stains compared to the reference strain GMI1000. The right panel shows the resulting alterations in the amino acid sequences of the T3E proteins

The composition of T3E repertoires often defines the host range of specific strains. In this regard, we have identified over 70 T3Es in each strain based on comparisons with effector sequences in public databases (Table 8). Comparisons with the reference GMI1000 strain suggest that the FJAT-91 strain lacks the T3E genes *ripAG*, *ripS4*, *ripM*, *ripP3*, *hyp16*, *ripAI* and *ripY*; the FJAT-452 strain lacks the T3E genes *ripP3*, *hyp16* and *ripM*, and the FJAT-462 strain lacks the T3E genes *ripAI*, *ripS4*, *ripP3*, *hyp16*, *ripM* and *ripAM*. On the other hand, several T3E genes that are not present in GMI1000 were found in the three newly sequenced strains, including *ripBE* (in FJAT-462), *ripS7* (in FJAT-452), *hyp7* (in FJAT-452 and FJAT-462) and *ripAL* and *ripF2* (in all 3 strains). The presence of most new T3E genes was confirmed by sequence analysis of PCR-amplified fragments from the three strains, being 100% identical among them and very similar or identical (78.84–100%) to their closest orthologs from other sequenced strains (Fig. 3). However, the *hyp7* gene from FJAT-462 has a 1206 bp insertion annotated as a transposase 180 bp downstream the start codon (Fig. 3). By comparing the sequences of the T3E genes that are shared by the three newly sequenced strains and the reference strain GMI1000, we identified 652, 798 and 692 variant sites in T3E sequences of FJAT-91, FJAT-452, FJAT-462, respectively (Table 9). These variations were classified into 7 types: missense variant, synonymous variant, frame shift variant, inframe deletion, inframe insertion, stop codon gain, and stop codon loss. Among them, 351 variations are shared by the three newly sequenced strains (Fig. 4). For example, the effector *ripA1* has both missense and synonymous variants, *ripAZ1* have a frame-shift variant, and *ripX* has an inframe deletion in all three strains (Fig. 5).

**Table 8:** Annotation and comparison of Type III effector genes in the three newly sequenced strains

	FJAT-91	FJAT-452	FJAT-462
Number of T3E genes by Prokka annotation	52	62	60
Total T3E genes after homology search	72	78	75
Number of T3E genes not present in GMI1000	2	4	4
Annotation of T3E genes not present in GMI1000	*ripAL* *ripF2*	*ripAL* *ripF2* *ripS7* *hyp7*	*ripAL* *ripBE* *ripF2* *hyp7*
Number of T3E genes in GMI1000 but not found in newly sequenced strain	7	3	6
Annotation of T3E genes in GMI1000 but not found in newly sequenced stain	*ripAG* *ripAI* *ripM* *ripP3* *ripS4* *ripY* *hyp16*	*ripM* *ripP3* *hyp16*	*ripAI* *ripAM* *ripM* *ripP3* *ripS4* *hyp16*

**Table 9:** Numbers and types of sequence variations (SNPs and INDELs) identified in the Type III effector genes (SNPs and INDELs) between the three newly sequenced strains and the reference stain GMI1000

Type	FJAT-91	FJAT-452	FJAT-462
All variants	652	798	692
Missense variant	268	350	299
Synonymous variant	378	439	382
Frameshift variant	2	4	6
Inframe deletion	1	1	2
Inframe insertion	3	3	2
Stop codon gain	0	0	1
Stop codon loss	0	1	0

Besides T3Es, *R. solanacearum* employs several additional virulence factors to achieve infection, such as EPS. The signaling cascade leading to the production of EPS involves several different regulatory components [25]. We analyzed the presence of genes involved in the regulation of EPS production, and found that all the major regulators are present in the three strains (Table 7). These genes displayed a high percentage of similarity when compared to their homologs in the GMI1000 reference strain, with most genes ranging from 98.35%–100% at the DNA level (98.26–100% at the amino acid level), with the exception of *phcB*, which shows a lower similarity in the FJAT-91 and FJAT-452 strains (86.41% at the DNA level in both strains) (Table 7). Other genes encoding putative virulence factors, such as *egl* (encoding an endoglucanase) and *pehB* (encoding an exo-poly-α-d-galacturonosidase) were also present in the three strains, with >99% similarity at the DNA and amino acid level compared to GMI1000 (Table 7).

## Conclusions

Earlier studies on the T3E repertoires of different plant pathogens suggested that T3E composition might shape the host range [6, 7]. In this study, we sequenced and analysed the genome of three *R. solanacearum* strains isolated from different host plants with similar geographical origin (Fujian province, China). Our analysis indicates that each one of these strains have a unique effector repertoire (Table 7). In contrast to what we observed for T3E genes, all the analysed genes involved in the perception of plant signals and the regulation of virulence factors were present in all strains, and displayed a high degree of similarity between the newly sequenced strains and the GMI1000 reference strains (Table 7), suggesting that the mechanism of perception of plant signals does not differ significantly among bacteria infecting different plant species.

In addition to their presence or absence in specific strains, T3E genes may undergo several types of mutations that change or disrupt their coding sequence. As a consequence, the encoded proteins may lose the original function, become unstable, or gain a new function. This allelic diversification may be imposed by the host defense system, and allows pathogens to avoid perception by the immune system of resistant host plants, in a phenomenon called pathoadaptation [26]. We identified alterations in effector sequences that were conserved in the three sequenced strains (Fig. 4). These sequence modifications may be due to the geographical distribution of these strains in comparison with the GMI1000 reference strain, originally isolated from French Guyana (South America), and may have functional relevance in the subversion of host functions in specific environmental conditions. Similarly, it is noteworthy that the FJAT-91 strain lacks 7 T3Es compared to GMI1000, while both are able to cause disease in tomato plants. Comparative analyses using the same tomato cultivars in controlled conditions will determine whether (i) these effectors are really dispensable to infect tomato, (ii) these effectors are dispensable in specific tomato cultivars, (iii) these effectors trigger immunity in specific tomato cultivars, or (iv) the environmental conditions in the FJAT-91 isolation site are more favourable to *R. solanacearum* infection, rendering unnecessary their virulence activities. The strain-specific absence of T3E genes or strain-specific loss-of-function variants (Fig. 4) may be caused by adaptation of these strains to specific hosts. Similarly, the transposase insertion in *hyp7* (specific from FJAT-462; Fig. 3) is likely to alter or abolish the function of the encoded T3E in this strain, and may suggest that this T3E is not needed (or its alteration is actually required) to cause disease in chili pepper plants. Additional functional characterization will be required to determine whether these effectors induce immune responses in eggplant or chili pepper, and may allow the identification of novel sources of resistance against *R. solanacearum* *.* Our analysis shows that these unique effector repertoires are sufficient to cause disease in different hosts within a similar geographical location, allowing us to reduce the impact of environmental conditions in the analysis of the requirement of T3Es to cause infection. This information, together with the increasing number of sequenced *R. solanacearum* strains, constitutes one more step towards the identification of host specificity determinants for *R. solanacearum*.

## Abbreviations

BLAST: Basic local alignment search tool
CDS: Coding sequences
COG: Clusters of orthologous groups
EPS: Exopolysaccharide
E-value: Expect value
INDEL: Insertion-deletion
ncRNAs: Noncoding RNAs
Pfam: Protein families
SNP: Single nucleotide polymorphism
T3E: Type-III effector
T3SS: Type-III secretion system
